# Dental stem cell-derived extracellular vesicles as promising therapeutic agents in the treatment of diseases

**DOI:** 10.1038/s41368-021-00152-2

**Published:** 2022-01-04

**Authors:** Ye Li, Xu Duan, Yinxue Chen, Bingyun Liu, Gang Chen

**Affiliations:** 1 The State Key Laboratory Breeding Base of Basic Science of Stomatology (Hubei-MOST) & Key Laboratory of Oral Biomedicine Ministry of Education, School and Hospital of Stomatology, Wuhan University, Wuhan, China; 2 Department of Oral and Maxillofacial Surgery, School and Hospital of Stomatology, Wuhan University, Wuhan, China; 3 Frontier Science Center for Immunology and Metabolism, Wuhan University, Wuhan, China

**Keywords:** Mesenchymal stem cells, Extracellular signalling molecules

## Abstract

Dental stem cells (DSCs), an important source of mesenchymal stem cells (MSCs), can be easily obtained by minimally invasive procedures and have been used for the treatment of various diseases. Classic paradigm attributed the mechanism of their therapeutic action to direct cell differentiation after targeted migration, while contemporary insights into indirect paracrine effect opened new avenues for the mystery of their actual low engraftment and differentiation ability in vivo. As critical paracrine effectors, DSC-derived extracellular vesicles (DSC-EVs) are being increasingly linked to the positive effects of DSCs by an evolving body of in vivo studies. Carrying bioactive contents and presenting therapeutic potential in certain diseases, DSC-EVs have been introduced as promising treatments. Here, we systematically review the latest in vivo evidence that supports the therapeutic effects of DSC-EVs with mechanistic studies. In addition, current challenges and future directions for the clinical translation of DSC-EVs are also highlighted to call for more attentions to the (I) distinguishing features of DSC-EVs compared with other types of MSC-EVs, (II) heterogeneity among different subtypes of DSC-derived EVs, (III) action modes of DSC-EVs, (IV) standardization for eligible DSC-EVs and (V) safety guarantee for the clinical application of DSC-EVs. The present review would provide valuable insights into the emerging opportunities of DSC-EVs in future clinical applications.

## Introduction

Localized in various tissues, such as bone marrows, umbilical cords, muscles, and fats, mesenchymal stem cells (MSCs) hold great potential for the treatment of different diseases.^1–3^ Dental stem cells (DSCs) are important sources of MSCs, they can be easily obtained by less invasive procedures and used with less ethical concerns.^4,5^ Being capable of regenerating not only dental tissues, but also other somatic tissues, DSCs have recently attracted great attention as promising tools for regenerative therapy.^6–9^

**Fig. 1:**
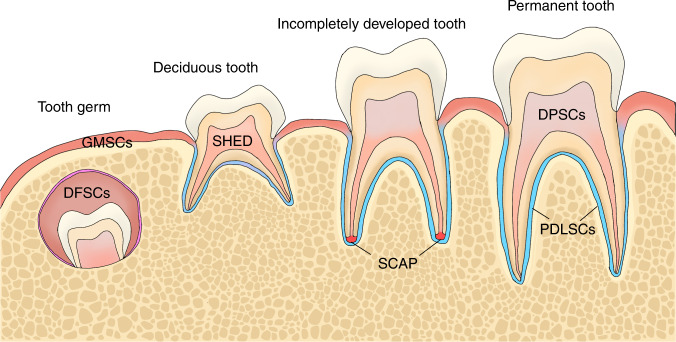
Different populations of dental stem cells (DSCs). DFSCs are derived from the developing tooth germ and can be isolated from the connective tissue around the tooth. SHED are harvested from the dental pulp of exfoliated deciduous tooth. SCAP come from the apical papilla of an incompletely developed tooth. In the permanent tooth, DPSCs and PDLSCs can be isolated from dental pulp and periodontal ligament. GMSCs exist in the gingiva around the tooth. DFSCs, dental follicle stem cells; SHED, stem cells from human exfoliated deciduous teeth; SCAP, stem cells from apical papilla; DPSCs, dental pulp stem cells; PDLSCs, periodontal ligament stem cells; GMSCs, gingival mesenchymal stem cells; DSCs, dental stem cells

To date, multiple subpopulations of DSCs have been investigated (Fig. 1), including dental pulp stem cells (DPSCs),^10^ periodontal ligament stem cells (PDLSCs),^11^ gingival mesenchymal stem cells (GMSCs),^12^ dental follicle stem cells (DFSCs),^13^ stem cells from human exfoliated deciduous teeth (SHED)^14^ and stem cells from apical papilla (SCAP).^15^ Although the effectiveness of DSC-based therapy has long been well recognized, mechanisms underlying the therapeutic actions remain elusive. Early studies indicated that DSCs functioned through cell differentiation after their targeted migration to the injury site, while emerging studies have revealed the low engraftment of transplanted DSCs and challenged the established dogma.^16^ The contribution of DSCs to disease treatment is increasingly ascribed to an indirect paracrine manner.^17^ By secreting a broad of secretomes, DSCs are capable of modulating the action of recipient cells locally and distantly.^18^ A variety of soluble factors, such as growth factors, chemokines, and cytokines, have been involved in the paracrine effects of DSCs.^19,20^ Recently, extracellular vesicles (EVs) have gained more attention as potential paracrine effectors in DSC-based therapy.

**Fig. 2:**
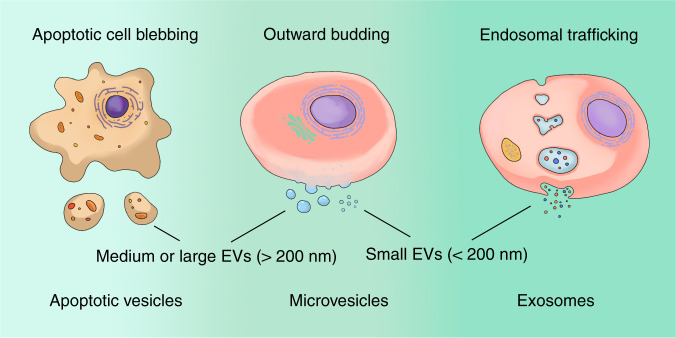
Classic assorting populations and updated classifications of DSC-derived extracellular vesicles (DSC-EVs). Traditionally, the populations of DSC-EVs could be categorized as apoptotic vesicles (disassembled from apoptotic cells), microvesicles (formed by direct outward budding), and exosomes (assembled through endosomal trafficking) according to their biogenesis mechanisms. The International Society for Extracellular Vesicles (ISEV) updated EV classification in 2018 and categorized EV subtype by small EVs (<200 nm) and medium/large EVs (>200 nm) in terms of size distribution

EVs are membrane-enclosed particles naturally released from theoretically all types of cells including DSCs (Fig. 2).^21,22^ According to the biogenesis and release pathways, EVs are conventionally classified into apoptotic vesicles (apoVs), microvesicles (MVs), and exosomes.^23^ By selectively carrying the “eat-me” signal phosphatidylserine, apoptotic cell disassembled apoVs often serve as critical modulators in cell clearance.^24^ A recent study also revealed the regulatory roles of apoVs in macrophage homeostasis, which extended the functional understanding of apoVs.^25^ MVs and exosomes are the more investigated EVs. MVs are formed by direct outward budding of the cell plasma membrane,^26^ while exosomes are generated through the endosomal pathway.^27^ Being enriched with biological cargos, MVs and exosomes are considered as potential biomarkers of diseases as well as important agents in disease treatments.^28,29^ Besides the classic categories of EVs, the International Society for Extracellular Vesicles (ISEV) updated the latest EV classification in 2018 in terms of the size distribution. EVs were assorted by small EVs (< 200 nm) and medium or large EVs (>200 nm).^30^

It has been documented that DSCs release numerous EVs (DSC-EVs).^31^ Encapsulating and transporting critical bioactive factors (e.g., proteins and nucleic acids) to modify the phenotype of target cells, DSC-EVs were effective in the treatment of multiple diseases, included but not limited to oral and craniofacial diseases. A better insight into the therapeutic potential of DSC-EVs would provide more reasonable options for the future treatment of certain diseases. Herein, we summarize the latest in vivo evidence that supports the therapeutic potential of DSC-EVs, providing up-to-date knowledge of the current status of DSC-EVs. Moreover, several keys to facilitate the clinical translation of DSC-EVs are also prospected.

## DSC-EVs as potential saviors for the treatment of diseases

### Dental diseases

Endodontic inflammation and periodontal diseases destruct pulp-dentin complex and periodontal supporting tissues irreversibly. Derived from dental tissues, DSC-EVs are considered naturally potentiated for pulp/dentin and periodontal regeneration (Table 1).

**Table 1:** In vivo therapeutic effects of DSC-EVs in oral diseases

Donor cells	Animal models	Therapeutic effects	Potential molecular mechanism	References
DPSCs	Tooth root slice model in nude mice	Pulp regeneration	Promoting odontogenic differentiation of DPSCs via triggering P38 phosphorylation	^32^
	Dental pulpotomy model in rats	Pulp regeneration	Inducing macrophage M2 polarization by delivering miR-125a-3p	^38^
	Periodontitis model in mice	Periodontal regeneration	Promoting macrophage M2 polarization via transferring miR-1246	^47^
	Pulpotomy model in rats	Dentine regeneration	Not mentioned	^43^
	Pulpless root canal model in rats	Pulp regeneration	Not mentioned	^41^
	Dental pulp injury model in miniature pigs	Dentin regeneration	Not mentioned	^45^
SHED	Tooth root slice model in nude mice	Pulp regeneration	Enhancing angiogenesis by transferring miR-26a that activated TGF-β/SMAD2/3	^34^
	Periodontitis model in mice	Periodontal regeneration	Not mentioned	^48^
SCAP	Tooth root slice model in nude mice	Dentine regeneration	Not mentioned	^44^
GMSCs	Periodontitis model in mice	Periodontal regeneration	Delivering miR-1260b to inhibit the osteoclastogenic activity of periodontal ligament cells by targeting the Wnt5a-mediated RANKL pathway	^49^

### Pulp regeneration

Dental pulp is a complex structure containing heterogeneous cell populations, such as DPSCs, endothelial cells, and immune cells. These populations serve as key contributors to pulp regeneration. The crucial challenge for effective pulp regeneration is to regulate these cell populations to achieve a comprehensive action. Of note, current findings have clarified the critical roles of DSC-EVs in manipulating DPSCs, endothelial cells, and macrophages in vivo. With a tooth root slice model implanted in nude mice, Huang et al. revealed the pulp regeneration effects of DSC-EVs through regulating DPSCs. They observed the lipid raft/caveolae-dependent endocytosis of DSC-EVs by DPSCs and revealed that DSC-EVs induced the odontogenic differentiation of DPSCs by triggering P38 phosphorylation.^32^

DSC-EVs may also enhance the angiogenic potential of endothelial cells in the process of pulp regeneration. Angiogenesis is a physiological process to rebuild adequate vasculatures and blood supply that is essential for successful pulp regeneration.^33^ Using a tooth fragment model in immunocompromised mice, Wu et al. revealed the pro-angiogenic capacities of DSC-EVs after 12 weeks of transplantation. With microRNA (miRNA) sequencing, miR-26a was identified as the therapeutic effector. In the recipient endothelial cells, DSC-EVs and their delivered miR-26a may function through regulating the angiogenic TGF-β/SMAD2/3 pathway.^34^ Of interest, researchers have also indicated that bone marrow mesenchymal stem cell-derived EVs (BMMSC-EVs) possess pro-angiogenic effects, but they may function through miR-21 and miR-1246, being different from DSC-EVs.^35^

Macrophages are considered critical regulators in the resolution of inflammation-related diseases including pulpitis.^36^ During the process of pulp regeneration, the reactions of macrophages create a regulatory microenvironment for odontogenesis.^37^ In response to pulp inflammation, macrophages would polarize to M1 (pro-inflammatory) and M2 (anti-inflammatory) phenotype. Induction of the pro-healing M2 macrophages was important for pulp repair and regeneration. In a pulp exposure model of rat, DSC-EVs remarkably switched macrophage polarization to the M2 phenotype and promoted pulp regeneration. Of note, miR-125a-3p was mechanistically considered as the effector by inhibiting TLR and NF-κΒ, critical mediators of inflammatory response.^38^ Interestingly, another study indicated that miR-124-3p in BMMSC-EVs potentially mediated macrophage polarization,^39^ suggesting the difference of therapeutic miRNAs between BMMSC-EVs and DSC-EVs.

Mechanistically, DSC-EVs transferred therapeutic molecules, especially miRNAs, to recipient cells to promote pulp regeneration. In recipient cells, DSC-EVs and their transferred miRNAs may function through triggering important signaling pathways. However, how these functional miRNAs were sorted into DSC-EVs from their donor cells and what factor would impact the sorting process remains largely unknown. Notably, Hu et al. revealed that the miRNA profiles within DSC-EVs greatly changed when their donor cells were cultured in an odontogenic medium.^40^ Importantly, the changed miRNA profiles especially miR-27a-5p led to an improved effect of DSC-EVs on odontogenesis, which is essential for pulp regeneration. Similarly, Chen et al. found that lipopolysaccharide (LPS) preconditioning also enhanced the pulp regenerative potential of DSC-EVs in rats.^41^ These findings suggest that appropriate precondition may enhance the therapeutic potential of DSC-EVs in pulp regeneration by sorting more effective miRNAs. Revealing the molecular mechanisms behind would facilitate future translation of DSC-EVs.

To date, there are limited in vivo studies directly demonstrating the pulp regenerative effects of other types of MSC-EVs, suggesting the potential unique role of DSC-EVs in pulp regeneration. Of interest, Murakami et al. revealed that, compared to the conditioned medium (CM) from DSCs, the CM from BMMSCs and adipose stem cells (ASCs) presented limited pro-angiogenic and odontogenic potential,^42^ indirectly suggesting a unique pulp regenerative effect of DSC-secretomes, including DSC-EVs. In this regard, a comparative study between DSC-EVs and other MSC-EVs in pulp regeneration are encouraged in the future to provide valuable insights into the unique therapeutic potential of DSC-EVs.

### Dentin regeneration

Dentin formation as a defensive reaction is of great importance in endodontic repair. Swanson et al. proposed a novel amphiphilic synthetic polymeric vehicle, which was able to control the long-term release of DSC-EVs. Tertiary dentine formation was observed after 6 weeks of transplantation into the defect area in a rat molar pulpotomy model.^43^ Zhuang et al. also embedded DSC-EVs in the root fragment enriched with BMMSCs and observed dentine formation after 12 weeks of subcutaneously transplantation in nude mice.^44^ Besides the rodent models, DSC-EVs combined with treated dentin matrix (TDM) also presented the potential of dentin formation in a pulp exposure model of miniature pig, suggesting DSC-EVs loaded in TDM as a promising strategy for pulp-capping therapy.^45^ Despite the obvious effects in vivo, the detailed molecular action of DSC-EVs in dentinogenesis remains unclear, calling for further mechanistic studies.

### Periodontal regeneration

Periodontitis is a worldwide inflammatory disease that leads to the loss of tooth support. Similar to their roles in pulpitis, macrophages are also critically involved in the destruction of periodontium.^46^ By converting the phenotype of macrophages from M1 to M2, DSC-EVs suppressed periodontal inflammation in mice.^47^ It was further elucidated that miR-1246 within DSC-EVs accounted for the therapeutic effect, also by targeting the p38 MAPK pathway. Wei et al. indicated that, by inhibiting the expression of the inflammatory cytokines and enhancing the osteogenic capabilities of BMMSCs, DSC-EVs were potentiated to treat periodontitis.^48^ In line with the precondition approach in pulp regeneration, studies also indicated that DSCs may secrete EVs with enhanced therapeutic potential in periodontal bone defects under disease-related stimulations. Nakao et al. examined the effects of TNF-α preconditioned-DSC-derived EVs on mouse periodontal maxillary bone loss and revealed that the up-regulated miR-1260b within DSC-EVs accounted for the enhanced prevention potential. Mechanistically, miR-1260b inhibited the osteoclastogenic activity of periodontal ligament cells by targeting Wnt5a-mediated RANKL pathway.^49^ Paradoxically, in the same study, LPS stimulation on DSCs did not cause any change on the effect of their derived EVs and the miRNA profiles within, requiring further investigations on the discrepancy.

### Systemic diseases

In addition to dental diseases, studies also confirmed the regenerative potential of DSC-EVs in systemic diseases, such as craniofacial bone defects, neurological disorders, wound injuries, and immune-related conditions (Table 2).

**Table 2:** In vivo therapeutic effects of DSC-EVs in systemic diseases

Donor cells	Animal models	Therapeutic effects	Potential molecular mechanism	References
DPSCs	Mandible defect model in rats	Bone regeneration	Enhancing osteogenesis through insulin-MAPK signaling axis	^57^
	Full-thickness skin wound model in mice	Wound healing	Not mentioned	^75^
	Calvarial defect model in rats	Bone regeneration	Not mentioned	^56^
SHED	Carrageenan-induced paw edema model in mice	Anti-inflammation	Suppressing edema by inhibiting the activities of cathepsin B and matrix metalloproteinases	^77^
	Traumatic brain injury model in rats	Motor function improvement	Not mentioned	^61^
	Parkinson’s disease model in rats	Motor function improvement	Not mentioned	^64^
	Systemic lupus erythematosus (SLE) model in mice	Immune regulation in SLE	Treating SLE by the delivery of miRNAs that target telomerase activity	^79^
PDLSCs	Autoimmune encephalomyelitis model in mice	Anti-inflammation	Inducing anti-inflammatory and immunosuppressive effects by inhibiting pro-inflammatory cytokines and promoting anti-inflammatory cytokines	^78^
	Calvarial defect model in rats	Bone regeneration	Not mentioned	^51^
	Calvarial defect model in rats	Bone regeneration	Inducing vascularization via increasing the level of VEGF	^54^
SCAP	Full-thickness circular gingival wound model in mice	Wound healing	Inducing vascularization by transferring Cdc42	^76^
GMSCs	Diabetic skin defect model in rats	Wound healing	Not mentioned	^73^
	Full-thickness gingival wound model in mice	Wound healing	Promoting wound healing by releasing IL-1RA contained EVs	^74^
	Calvarial defect model in rats	Bone regeneration	Not mentioned	^52^
	Calvarial defect model in rats	Bone regeneration	Promoting osteoangiogenesis by delivering miR-2861 and miR-210	^53^
	Sciatic nerve injury model in mice	Peripheral nerve regeneration	Promoting proliferation and migration of Schwann cells through upregulating c-JUN/JNK pathway	^65^
	Sciatic nerve injury model in rats	Peripheral nerve regeneration	Not mentioned	^66^
	Tongue defect model in rats	Taste bud regeneration	Inducing the differentiation of epithelial basal progenitor cells into taste bud cells by increasing the expression of BDNF	^69^

### Craniofacial bone defects

Repair of critical-sized calvarial defects caused by congenital abnormalities or trauma has long been considered a paramount challenge.^50^ Diomede and colleagues constructed a platform with DSC-EVs and three-dimensional engineered scaffolds and induced calvarial bone regeneration in rats.^51,52^ Mechanistic speculation attributed the fundamental roles of DSC-EVs to the miRNA cargos, mainly miR-2861 and miR-210, for their potential effects on osteoangiogenesis.^53,54^ However, Chen et al. indicated a different effective cargo (miR-375) within ASC-EVs that was responsible for the calvarium reconstruction in rats.^55^ When comparing the therapeutic effects of DSC-EVs with their donor cells in a calvarial bone defect model in rat, it was indicated that DSC-EVs showed almost the same potential as DSCs, supporting DSC-EVs as alternatives in treating calvarium defects.^56^ Progress has also been made for DSC-EVs in the treatment of mandible defects in rodent models. Jin et al. identified the insulin–MAPK axis as the key contributor to the mandible regeneration induced by DSC-EVs.^57^

### Neurological disorders

Attributing to the neural crest origin, DSCs and their derived EVs presented remarkable neuro-regeneration potential, both in the central and peripheral nervous system.^58^ Microglial cells are the major cellular components of the innate immune system of the central nervous system (CNS).^59^ Similar to macrophages, microglial cells also have two different phenotypes. The M1 phenotype is related to the promotion of brain injury, while the M2 phenotype is responsible for CNS repair.^60^ In our previous study, DSC-EVs recovered impaired motor function in rat traumatic brain injury (TBI) models by inducing M2 microglia polarization, suggesting DSC-EVs as new remedies in TBI and other microglia-mediated CNS diseases.^61^ Spinal cord injury (SCI) is another devastating condition that results in neurological dysfunction and paralysis. Asadi-Golshan et al. revealed that the injection of DSC-CM-loaded hydrogel promoted function recovery of rats with SCI, suggesting the therapeutic potential of DSC-secretomes, possibly via their derived EVs.^62^ To further investigate the effects of DSC-EVs on SCI, Guo et al. incubated neurons with DSC-EVs in vitro. Robust axonal outgrowth was achieved with DSC-EV treatment, indicating a potential in vivo mechanism of action with DSC-EVs.^63^ Moreover, intranasal administration of DSC-EVs also improved motor function of rats with Parkinson’s disease by rescuing the tyrosine hydroxylase expression in the striatum and substantia nigra.^64^ Till now, the mechanism behind the neuroprotective action of DSC-EVs remains elusive. Anti-oxidative proteins contained in DSC-EVs may partially account for their therapeutic effects, however, further mechanistic investigations are in great need to provide valuable insights into the therapeutic interventions for CNS diseases.

DSC-EVs were also reported to induce peripheral nerve regeneration. It has been critically considered that by promoting proliferation and migration of Schwann cells through upregulating c-JUN/JNK pathway, DSC-EVs succeed in regenerating sciatic nerve in rodent models.^65,66^ The c-JUN/JNK signaling pathway was reported to be critically involved in the protection of cerebral neurovascular inflammation.^67^ Besides neurological disorders, extensive reports have shown the protective roles of c-JUN/JNK pathway in metabolic diseases, such as obesity and type 2 diabetes, suggesting a potential therapeutic effect of DSC-EVs in related diseases.^68^ Taste buds are peripheral sensors located on the surface of the tongue, by embedding DSC-EVs in small intestinal submucosa extracellular matrix and transplanting them into tongue defects of rats, Zhang et al. confirmed the potential of DSC-EVs in repairing the tongue epithelium papillae and regenerating the taste bud.^69^ It was further indicated that DSC-EVs induced the differentiation of epithelial basal progenitor cells into taste bud cells by increasing the level of BDNF, one of the most studied neurotrophins that could benefit the treatment of neurodegenerative diseases.^70^ Inducing BDNF, DSC-EVs may also serve as a future therapeutic approach in brain degenerative disorders, such as Alzheimer’s disease and Parkinson’s disease. Of interest, Venugopal et al. compared the neuroprotective potential of DSC-EVs and BMMSC-EVs and revealed the increased level of endogenous BDNF in DSC-EV treatment group than BMMSC-EVs, suggesting a potential better neurological therapeutic effect of DSC-EVs.^71^

### Wound injuries

In general, oral gingival wounds heal faster with less scar.^72^ However, the unique mechanism behind is elusive. Hence, studies investigated the effects of DSC-EVs from gingiva tissues in the events of wound healing. Evidence indicated that DSC-EVs from gingiva tissues played positive roles in motivating the wound healing of diabetic rats.^73^ To move forward, researchers further interpreted the mechanism behind the faster healing potential of DSC-EVs. By cytokine array analysis on DSC-EVs and skin MSC-EVs, Kou et al. identified IL-1RA, a natural inhibitor of the proinflammatory cytokine IL-1β, as the critical factor. Carrying higher amounts of IL-1RA than skin MSC-EVs, DSC-EVs from gingiva tissues presented accelerated wound healing effect in mice.^74^ Intriguingly, Fas/Fap-1/Cav-1 regulated IL-1RA release in DSCs by binding to SNAP25/VAMP5. Moreover, TNF-α mediated the IL-1RA secretion by upregulating the expression of Fas/Fap-1 and inducing the membrane translocation of Fas/Cav-1.

Besides the direct delivery of healing molecules, DSC-EVs also displayed the therapeutic potential by enhancing vessel formation, similar to their action during pulp regeneration.^75^ In this case, DSC-EVs transferred Cdc42, the critical player in cytoskeletal reorganization, to promote the proliferation and migration of endothelial cells for inducing vascularization in wound healing.^76^ Containing both angiogenic proteins and miRNAs (e.g., miR-26a), DSC-EVs may facilitate angiogenesis-related injuries that are not limited to wounds and pulp diseases.

### Immune-related conditions

DSC-EVs present immune-modulatory potential in multiple manners. By directly transferring annexin A1, a critical regulator in innate and adaptive immunity, DSC-EVs suppressed carrageenan-induced acute inflammation in mice.^77^ Indirectly, DSC-EVs also presented immunosuppressive effects in a mouse model of experimental autoimmune encephalomyelitis (EAE) by inhibiting pro-inflammatory cytokines and promoting anti-inflammatory cytokines.^78^ Mechanistic speculations held that the effective role of DSC-EVs were owed, at least partially, to the RNAs inside. In consistent, the therapeutic effects of DSC-EVs pretreated with RNase were significantly attenuated in the mouse models of systemic lupus erythematosus.^79^

### Toward future translation of DSC-EVs: keys to progress

**Fig. 3:**
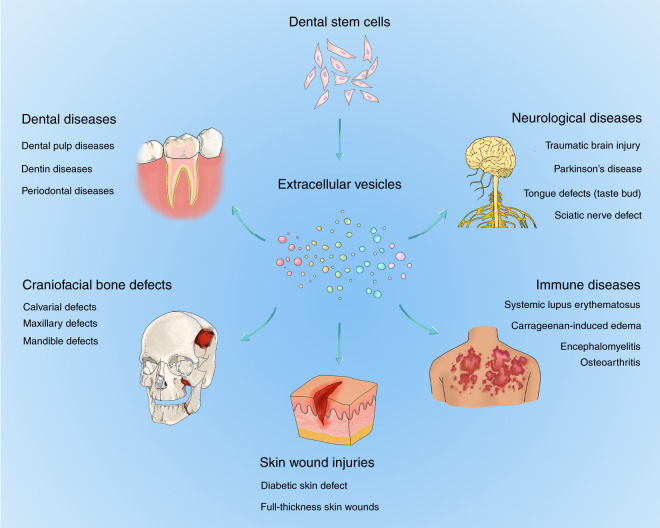
In vivo therapeutic potential of DSC-EVs. Briefly summarized therapeutic potential of DSC-EVs were shown, mainly in dental diseases, craniofacial bone defects, neurological disorders, skin wound injuries, and immune-related conditions

**Fig. 4:**
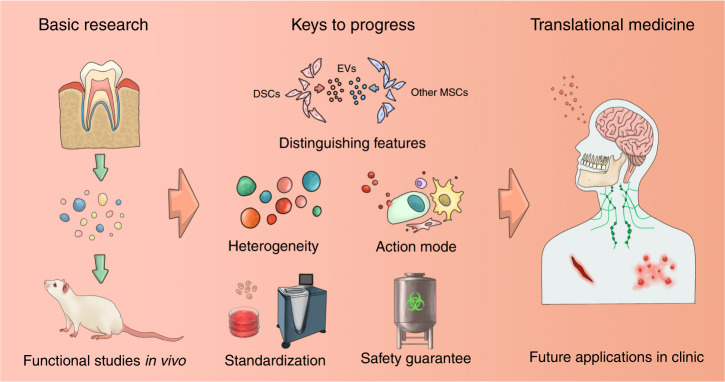
Keys for DSC-EVs to progress in future translational medicine. Although in vivo evidence indicated the promising effects of DSC-EVs in various diseases, key points should also be considered to facilitate future clinical settings. Firstly, the distinguishing features of DSC-EVs compared with other stem cell-derived EVs should be revealed for suggesting more appropriate roles of DSC-EVs in specific diseases. Secondly, the heterogeneity of DSC-EVs from different origins should be clarified to explain the distinguished therapeutic effects of DSC-EVs and provide better choice for specific diseases. Thirdly, revealing the action modes of DSC-EVs, from functional cargo packaging to recipient cell selecting would benefit DSC-EVs in targeted treatments and advance the potential application. Fourthly, standardization for eligible production of DSC-EVs should be optimized to meet the need for achieving the clinical level of DSC-EVs. Finally, safety guarantee of DSC-EVs are needed to take into serious consideration before clinical applications

Accumulating in vivo evidence indicated that DSC-EVs presented promising effects in the treatment of diseases (Fig. 3), while the manipulation of DSC-EVs to facilitate future translational medicine remains a challenge (Fig. 4). Crucial barriers, as well as potential solutions to foster the clinical application of DSC-EVs, are summarized.

### Distinguishing features of DSC-EVs compared with other types of MSC-EVs

As a subset of MSC-EVs, DSC-EVs carry both stem cell markers and EV markers. It has been revealed that DSC-EVs may serve as critical contributors to the treatment of numerous diseases. However, accumulating evidence also indicates that, compared to other types of MSC-EVs, DSC-EVs may possess better therapeutic potential in dental diseases, neurological disorders, and wound injury models. This may be owed to the origin of certain EVs. Derived from the neural crest, DSCs tend to be more differentiated into dental and neural tissue,^80^ which determines the inherited therapeutic cargos in their secreted EVs. Carrying and transferring specific therapeutic effectors to the target cells including DPSCs, endothelial cells and macrophages/microglial cells, DSC-EVs are potent in promoting odontogenesis, angiogenesis, and anti-inflammation, which are of great importance during the treatment of dental diseases, neurological disorders, and wound injuries. Indeed, documentations have reported the different contents between DSC-EVs and other types of MSC-EVs. Besides the differential therapeutic miRNAs between DSC-EVs and other MSC-EVs in certain disease models,^52–54^ it was also noted that the PIWI-interacting RNAs (piRNAs) were differentially enriched in DSC-EVs compared to BMMSC-EVs.^81^ Of interest, most of the enriched piRNAs in DSC-EVs were related to dental embryology and neuronal communication, providing a potential explanation on the unique role of DSC-EVs in dental diseases and neurological disorders. Nevertheless, existing evidence was still limited for a comprehensive comparison of the features between DSC-EVs and other types of MSC-EVs. To reveal more distinguishing features of DSC-EVs, extra efforts should be made for the full picture of discrepancy.

### Heterogeneity among different subtypes of DSC-derived EVs

Current knowledge on the therapeutic potential of DSC-EVs is based on EVs from certain kind of DSCs. The biological behavior of DSC-EVs obtained from distinct dental tissues may result in inconsistent therapeutic outcomes. Hence, unambiguous data of heterogeneity in all kinds of DSC-EVs should be highlighted to provide a better choice for specific diseases. With regards to this, several open questions should be discussed. Do DSCs from different origins hold similar or disparate potential for EV secretion? Is there any variability in the content or function among different subtypes of DSC-derived EVs? Is it possible to provide more appropriate DSC-EVs for specific disease treatments? To help answer these critical questions, special focus should be placed on the molecular mechanisms underlying the secretion of different subtypes of DSC-EVs. Meanwhile, high-throughput determinations should also be conducted to reveal the molecular profiles of different DSC-EV subtypes. Eventually, functional assays in vivo should be performed to provide direct evidence on the heterogeneity of DSC-EVs derived from different tissues.

### Action modes of DSC-EVs

Although supporting evidence has provided cues of bioactive components, especially miRNAs transferring from DSC-EVs to recipient cells. How the functional cargos are packaged into EVs and delivered to their recipient cells remains elusive. Revealing the molecular mechanisms on the sorting of critical cargos may not only be adopted for mechanistic insights into the role of DSC-EVs but also provide new targets on enhancing the therapeutic potential of DSC-EVs. In addition, considerations should also be emphasized on how DSC-EVs are directed toward the target tissues. Although the information on EV target selection has been provided partially,^82,83^ little is known of the unique action of stem cell especially DSC-derived EVs since their cell‐type specificity. In this context, appropriate labeling strategies should be developed for tracking the route of DSC-EV delivery as well as imaging the intercellular communication between DSC-EVs and recipient cells. A better understanding of the action modes of DSC-EVs would benefit future treatments, advancing the application of DSC-EVs to the next level.

### Standardization for eligible DSC-EVs

Despite the therapeutic potential of DSC-EVs in multiple diseases, their future clinical application is hampered by the unstable production and potency. Regarding this, standardized protocols for obtaining eligible DSC-EVs were in urgent need ahead of clinical translation. Donor cell culture is the first key step for producing eligible EVs. However, DSCs would lose their expansion capacity and therapeutic potential after a few passages in vitro.^84^ In addition, with increasing cell passage in culture, their derived EVs would lose parts of bioactivity due to the changes in protein and miRNA contents.^85^ Early passage (before P4) of DSC-EVs was thus recommended for eligible production. Besides, EVs secreted by different types of donor cells might contain different cargos, especially distinctive miRNA profiles, thus resulting in differential therapeutic effects.^86^ Hence, for certain disease treatment, selecting the most appropriate donor cell populations is critical for qualified DSC-EVs. Culture conditions, especially medium constituent may also lead to the compositional changes in DSC-EVs.^87^ For instance, odontogenic medium, LPS, and TNF-α precondition significantly changed the miRNA profiles within DSC-EVs and enhanced their therapeutic effect in certain diseases.^40,41,57^ Thus, optimized and standardized culture condition would benefit the reproducible therapeutic effects of DSC-EVs for future applications.

In addition to standardized condition for donor cell culture, technical standardization is also of central importance because the isolation, characterization as well as preservation, and transportation of EVs are all method-dependent considerations. To provide eligible DSC-EVs for clinical applications, efficient, reproducible, and clinical-friendly methodologies are required. To date, there are a variety of methods for EV isolation, such as ultracentrifugation, filtration, precipitation, and immunoaffinity techniques.^88,89^ Although each method presents advantages as well as disadvantages, ultracentrifugation-based techniques remain the gold standard for obtaining high-quality EVs as recommended by the ISEV.^30^ Although being able to isolate qualified EVs from a large volume of biological fluids, there are also limitations for ultracentrifugation-based techniques.^90^ The major drawback is the protein contaminations. To address this issue, techniques with combined ultracentrifugation and size exclusion chromatography are recently recommended to further improve the purity of the obtained EVs.^91^ Moreover, the characterization of DSC-EVs should also be focused regarding the differential impact of analytical parameters on the quality and quantity of EVs. To obtain EVs with standardized phenotypes, nanoparticle tracking analysis together with high-resolution flow cytometry are recommended for characterizing the key parameters of EVs (e.g., size, concentration, specific markers, and key components).^92^ For eligible DSC-EVs, it is also critical to preserve their stability and natural properties before clinical use. To prevent unexpected denaturation and degradation of the therapeutic cargos, −80 °C is suggested for the storage and transportation.^93^ Besides all the described concerns, aseptic operations should be strictly taken throughout the whole process. Also, the obtained DSC-EVs are recommended to be sterile-filtered through a 0.22 um filter for clinical use.^94^

### Safety guarantee for the clinical application of DSC-EVs

Guarantee of safety is indispensable for the clinical application of DSC-EVs. Achieving eligible DSC-EVs by standardized procedures, including the culture of donor cells, isolation, characterization, storage, and transportation of EVs would benefit the safety of DSC-EVs to a large extent. However, there are still several critical concerns to be considered. Containing various types of proteins and nucleic acids, DSC-EVs may not only lead to therapeutic effects, but also result in undesirable effects. The compositional analysis (e.g., protein and nucleic acid profiling) is thus essential for detecting unfavorable contents, such as oncogenes and toxic molecules.

Moreover, limitations on the general guidelines for the administration of EVs in vivo, taking pharmacokinetics and pharmacodynamics into account, have also hampered the safety guarantee of DSC-EVs. The route, dosage, and frequency of administration are all critical issues to be concerned. Systemic administration of EVs has been reported as the most common route of administration.^95,96^ However, the general distribution of the injected EVs in vital organs (e.g., lungs and liver) raises the potential biosafety issues when treating certain diseases such as wound injuries and bone defects.^97,98^ Regarding this point, feasible modification strategies should be developed to enhance the targeting ability of DSC-EVs. Alternatively, local administrations, such as local injection and intranasal delivery are recommended for specific diseases like bone-related diseases and neurological disorders.^99,100^ In addition, the inconsistent frequency and dosage of DSC-EVs are also considered as risk factors in the in vivo administration. Comprehensive assessments of dose-response kinetics in vivo are of great importance for setting the reasonable frequency of DSC-EV administration. Besides, dose quantification in animal models, as well as clinical trials, should be introduced in future studies to provide a suggested range for the safety guarantee of DSC-EVs. In 2020, the first DPSC product was approved by the National Medical Products Administration of China, providing a future outlook for this emerging and exciting field of product research on DSC and their derived EVs.

## Conclusion

Taken the supportive in vivo evidence together, DSC-EVs carried and transferred specific bioactive contents (e.g., functional RNAs and proteins) to target cells, presenting unique therapeutic potential in certain diseases. However, the future application of DSC-EVs is restricted by several crucial barriers. Continued efforts should be made to accelerate the clinical translation of DSC-EVs.
